# A more unstable resting-state functional network in cognitively declining multiple sclerosis

**DOI:** 10.1093/braincomms/fcac095

**Published:** 2022-04-12

**Authors:** Tommy A. A. Broeders, Linda Douw, Anand J. C. Eijlers, Iris Dekker, Bernard M. J. Uitdehaag, Frederik Barkhof, Hanneke E. Hulst, Christiaan H. Vinkers, Jeroen J. G. Geurts, Menno M. Schoonheim

**Affiliations:** 1 Department of Anatomy and Neurosciences, MS Center Amsterdam, Amsterdam Neuroscience, Amsterdam UMC, Vrije Universiteit Amsterdam, Amsterdam, The Netherlands; 2 Department of Neurology, MS Center Amsterdam, Amsterdam Neuroscience, Amsterdam UMC, Vrije Universiteit Amsterdam, Amsterdam, The Netherlands; 3 Department of Radiology and Nuclear Medicine, MS Center Amsterdam, Amsterdam Neuroscience, Amsterdam UMC, Vrije Universiteit Amsterdam, Amsterdam, The Netherlands; 4 Queen Square Institute of Neurology and Centre for Medical Image Computing, University College London, London, UK; 5 Department of Psychiatry, Amsterdam Neuroscience, Amsterdam UMC, Vrije Universiteit Amsterdam, Amsterdam, The Netherlands

**Keywords:** multiple sclerosis, network, connectivity, dynamic, cognition

## Abstract

Cognitive impairment is common in people with multiple sclerosis and strongly affects their daily functioning. Reports have linked disturbed cognitive functioning in multiple sclerosis to changes in the organization of the functional network. In a healthy brain, communication between brain regions and which network a region belongs to is continuously and dynamically adapted to enable adequate cognitive function. However, this dynamic network adaptation has not been investigated in multiple sclerosis, and longitudinal network data remain particularly rare. Therefore, the aim of this study was to longitudinally identify patterns of dynamic network reconfigurations that are related to the worsening of cognitive decline in multiple sclerosis. Resting-state functional MRI and cognitive scores (expanded Brief Repeatable Battery of Neuropsychological tests) were acquired in 230 patients with multiple sclerosis and 59 matched healthy controls, at baseline (mean disease duration: 15 years) and at 5-year follow-up. A sliding-window approach was used for functional MRI analyses, where brain regions were dynamically assigned to one of seven literature-based subnetworks. Dynamic reconfigurations of subnetworks were characterized using measures of promiscuity (number of subnetworks switched to), flexibility (number of switches), cohesion (mutual switches) and disjointedness (independent switches). Cross-sectional differences between cognitive groups and longitudinal changes were assessed, as well as relations with structural damage and performance on specific cognitive domains. At baseline, 23% of patients were cognitively impaired (≥2/7 domains *Z* < −2) and 18% were mildly impaired (≥2/7 domains *Z* < −1.5). Longitudinally, 28% of patients declined over time (0.25 yearly change on ≥2/7 domains based on reliable change index). Cognitively impaired patients displayed more dynamic network reconfigurations across the whole brain compared with cognitively preserved patients and controls, i.e. showing higher promiscuity (*P* = 0.047), flexibility (*P* = 0.008) and cohesion (*P* = 0.008). Over time, cognitively declining patients showed a further increase in cohesion (*P* = 0.004), which was not seen in stable patients (*P* = 0.544). More cohesion was related to more severe structural damage (average *r* = 0.166, *P* = 0.015) and worse verbal memory (*r* = −0.156, *P* = 0.022), information processing speed (*r* = −0.202, *P* = 0.003) and working memory (*r* = −0.163, *P* = 0.017). Cognitively impaired multiple sclerosis patients exhibited a more unstable network reconfiguration compared to preserved patients, i.e. brain regions switched between subnetworks more often, which was related to structural damage. This shift to more unstable network reconfigurations was also demonstrated longitudinally in patients that showed cognitive decline only. These results indicate the potential relevance of a progressive destabilization of network topology for understanding cognitive decline in multiple sclerosis.

## Teaser

Broeders *et al.* report that multiple sclerosis patients with cognitive impairment cross-sectionally show a more unstable resting-state functional network compared with patients with preserved cognition. Longitudinally, this network instability further progressed, but only in patients that demonstrated cognitive decline.

## Graphical Abstract

**Graphical Abstract:**
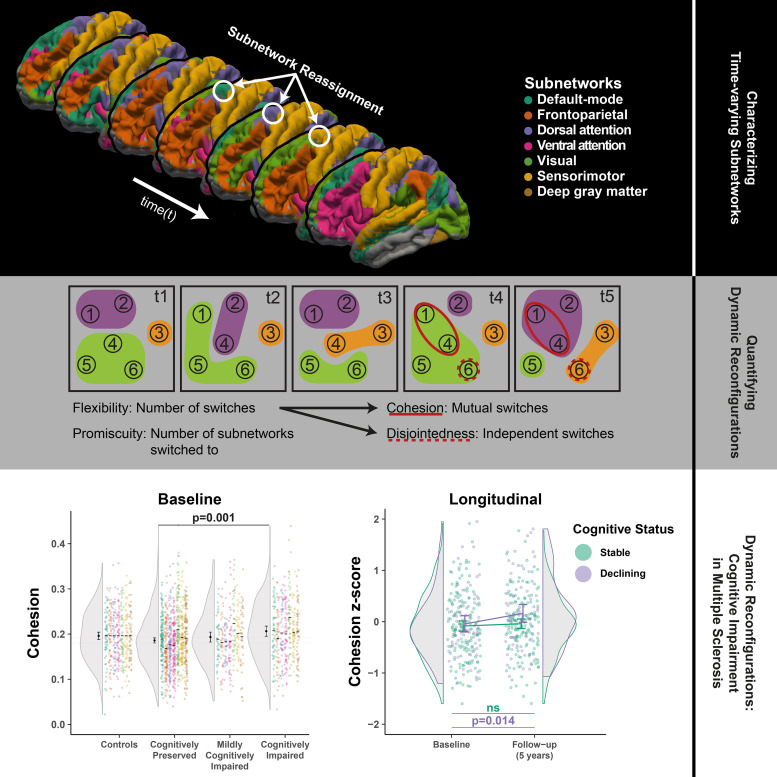

## Introduction

People with multiple sclerosis frequently suffer from cognitive impairment, which severely affects daily functioning.^1^ In multiple sclerosis, neuro-axonal damage occurs throughout the brain, and the structural brain network frequently becomes disconnected.^2^ The structural brain network characterizes the anatomical links between brain regions and represents the main pathways of communication between brain regions, whereas the functional network represents the presumed strength of communication over these anatomical pathways (i.e. functional connectivity),^3^ which can be characterized even in the absence of an explicit task (i.e. resting-state). In theory, accumulating structural network damage in multiple sclerosis could hamper effective integration of information across the brain and thereby affect normal cognitive functioning.

In health, the functional brain network as a whole is hierarchically organized into communities (i.e. subnetworks) of brain regions that are functionally coupled and differentially involved in specific cognitive processes.^4,5^ Previous work has shown that these subnetworks on average become structurally more segregated, starting in early stages of multiple sclerosis^6–8^ and worsening in later disease stages. This progressive disconnection between subnetworks has been related to worse cognitive function^9^ and could potentially be due to damage to the particularly vulnerable long-range connections necessary for integration of information between subnetworks. When examining the functional connectivity between brain regions in multiple sclerosis, some studies suggested more segregated subnetworks in cognitively impaired (CI) patients as well,^10,11^ while subnetworks like the default-mode and frontoparietal networks paradoxically become more strongly connected to the rest of the network.^12,13^ Thus, as structural disconnection worsens in these patients, functional connectivity seems to change in rather complex manner. There is ambiguity in interpreting these findings from the perspective of functional reorganization and assessing the balance between compensatory and maladaptive processes. In part, this may be caused by studying only time-averaged (i.e. static) functional connectivity; more recently studies have investigated the time-varying characteristics of the functional network. Such dynamic adaptation of the communication between (sub)networks can be investigated during a resting-state functional MRI (rs-fMRI) scan and the few studies applying dynamic functional imaging in multiple sclerosis have indicated that default-mode areas seem ‘locked’ in a highly connected state in patients with cognitive impairment.^14,15^ Another study showed that in CI multiple sclerosis patients, there is a reduction of switches between specific network conformations (i.e. states).^16^

These approaches have been highly valuable in establishing the concept of a more rigid or ‘stuck’ network in multiple sclerosis patients with cognitive impairment. However, a dynamic approach has not been used to specifically study the time-varying adaptation of subnetworks. These subnetworks are continuously and dynamically reconfigured in healthy individuals, especially when performing tasks that requires a higher level of integration of information across multiple subnetworks.^17,18^ Now, methodological advances allow for better characterization of these reconfigurations of subnetworks and make it possible to discern whether brain regions are reconfigured in unison or individually.^19,20^ Consequently, by investigating the brain from such a dynamic network perspective it becomes possible to quantify how information is integrated across subnetworks. This approach has, for example, shown that subnetworks became less stable in schizophrenia despite other reports showing that the network as a whole became more rigid,^21,22^ while such an approach has not been used in multiple sclerosis. It would, therefore, be interesting to investigate whether the effective integration of information across subnetworks is limited by reduced reconfigurations in multiple sclerosis. Alternatively, the network might actually become more unstable in multiple sclerosis, increasing reconfigurations. As such, this network concept could provide a new framework to describe functional network changes in multiple sclerosis and their impact on cognition.

Longitudinal studies are imperative to investigate whether dynamic network alterations relate to cognitive decline in multiple sclerosis, but such data remains rare. Therefore, the aim of this study was to investigate whether cognitive decline in multiple sclerosis is related to altered (cross-sectional and longitudinal) dynamic reconfiguration of subnetworks within the functional brain network. Dynamic network changes and cognitive performance were evaluated in rs-fMRI data from 230 patients with multiple sclerosis and 59 healthy individuals with two measurements at a 5-year interval. We hypothesized that multiple sclerosis patients with cognitive impairment would show reduced network adaptation (i.e. less reconfigurations) and that this would exacerbate over time in cognitively declining patients only.

## Materials and methods

### Participants

This study involves a retrospective analysis of prospectively attained longitudinal data from the Amsterdam multiple sclerosis cohort,^23,24^ including a total of 332 multiple sclerosis patients and 96 healthy controls (HCs) with available functional MRI data who were recruited between 2008 and 2012.^12–14,24–29^ Functional network dynamics in these participants was described previously.^14^ In total, 234 multiple sclerosis patients and 60 HCs returned for a 5-year follow-up between 2014 and 2017, of whom rs-fMRI and neuropsychological assessment was available at both time points for 230 patients (48 ± 11 years; 83 male) and 59 HCs (46 ± 10 years; 28 male). Approval was obtained from the local institutional ethics review board and written informed consent was provided by all participants. All patients were diagnosed with clinically definite multiple sclerosis according to the 2010 revised McDonald criteria,^30^ were relapse-free without steroid treatment for at least 2 months before participation and had no history of or current psychiatric and/or neurological disease besides multiple sclerosis. The Expanded Disability Status Scale (EDSS) was used to determine physical disability. Fatigue was determined in a subset of patients (*N* = 123) using the Checklist of Individual strength (CIS-20r), by summing all subdomain scores. Baseline rs-fMRI data of the Amsterdam multiple sclerosis cohort has previously been reported,^14^ but longitudinal rs-fMRI has not been investigated before.

### Neuropsychological evaluation and classification

Neuropsychological evaluation was performed on the same day as the MRI examination, using an expanded Brief Repeatable Battery of Neuropsychological tests^31^ as previously described.^32^ In short, performance on these tests was aggregated into seven cognitive domains and adjusted for age, sex and education based on the residuals of these variables in a matched HC cohort^33^ and transformed to *z*-scores at each time-point. Cognitive domains included executive functioning (concept shifting test), verbal memory (selective reminding test), information processing speed (IPS; symbol digit modalities test), verbal fluency (word list generation), visuospatial memory (spatial recall test), working memory (memory comparison test) and attention (Stroop colour-word test). The *z*-scores from these cognitive domains were averaged to produce a summary value of average cognition, which is only used to explore the relation between network dynamics and cognition and not to classify patient groups. Classification of CI patients was defined as scoring 2 standard deviations (SDs) or more below HCs on at least two cognitive domains.^12^ Patients that scored between 1.5 and 2 SDs below HCs on two or more cognitive domains were regarded as mildly cognitively impaired (MCI); all other patients were denoted as cognitively preserved (CP). The same approach was applied again to classify patients based on the follow-up data, these classifications were exclusively used for a validation analysis.^24^ Classification of longitudinal cognitive change has been described previously^23^ and was based on the practice-corrected reliable change index^34^ adjusted for the time-interval between baseline and follow-up. Patients with yearly change rates of more than 0.25 on two or more cognitive domains were considered cognitively declining and all others as cognitively stable.

### MRI acquisition

All scanning was performed using a 3 T whole-body MRI scanner (GE Signa-HDxt, Milwaukee, WI) with an 8-channel phased-array head coil. The scanner underwent a major upgrade between baseline and follow-up, which was corrected for using time-point specific *z*-scores based on the distribution of HCs for all longitudinal analyses, as reported previously.^24^ The scanning protocol included a 3D T_1_-weighted (3DT1) fast-spoiled gradient-echo sequence [repetition time (TR)/echo time (TE) = 7.8/3 ms; inversion time = 450 ms; flip angle = 12°; sagittal slice thickness = 1.0 mm; in-plane resolution = 0.9 × 0.9 mm], a 3D T_2_-weighted fluid-attenuated inversion recovery (FLAIR) sequence (TR/TE = 8000/125 ms; inversion time = 2350 ms; sagittal slice thickness = 1.2 mm; in-plane resolution =1.0 × 1.0 mm), a rs-fMRI echo planar imaging sequence (202 volumes; TR/TE = 2200/35 ms; flip angle = 80°; axial slice thickness = 3 mm, contiguous; in-plane resolution = 3.3 × 3.3 mm) and a diffusion tensor imaging sequence using five volumes without directional weighting (*b* = 0 s/mm^2^) and 30 with non-collinear diffusion gradients (*b* = 1000 s/mm^2^, TR/TE = 13000/91 ms, flip angle = 90°, axial slice thickness = 2.4 mm, contiguous; in-plane resolution = 2 × 2 mm).

### Image pre-processing

White matter lesion segmentation was performed on the FLAIR images,^35^ and masks were linearly registered to 3DT1-space for lesion filling.^36^ The rs-fMRI images were pre-processed with the MELODIC pipeline (FSL 5, fmrib.ox.ac.uk/fsl), including the removal of the first two volumes, motion correction, slice-time correction, brain extraction and 4 mm Gaussian smoothing. Subsequently, ICA-AROMA (v0.4-beta)^37^ was used for automatic removal of residual motion artefacts. Regression of mean white matter and cerebrospinal fluid signal, high-pass temporal filtering, boundary-based registration to 3DT1 images and co-registration and resampling to 4 mm Montreal Neurologic Institute (MNI-152) standard space was applied.

### Structural damage

Markers of structural damage in multiple sclerosis patients have previously been quantified for this cohort.^28^ In short, baseline deep grey matter volume was calculated using FIRST segmentations and normalized cortical grey matter volume by subtracting the FIRST segmentations from the SIENAX grey matter segmentation; both were normalized for head size. Lesion segmentations were used to determine white matter lesion volume. Diffusion tensor image processing as performed using FMRIB’s Diffusion Toolbox and included motion and eddy distortion correction, followed by diffusion tensor fitting. Fractional anisotropy (FA) was calculated for each voxel and non-linearly registered to the FMRIB58_FA template skeleton and the highest FA value perpendicular to all voxels of the skeleton were projected onto the skeleton. The average FA over the whole skeleton signified overall white matter integrity.

### Functional network analysis: atlas and subnetworks

All 210 cortical regions from the Brainnetome atlas^38^ were combined with 14 deep grey matter regions segmented using FIRST, which were transformed to standard space using inverted registration parameters of the 3DT1 scans. Voxels that represented white matter or cerebrospinal fluid (based on SIENAX segmentations) or showed distorted rs-fMRI signal were identified and excluded from the analysis.^12^ Regions that had <30% residual coverage after this step was discarded (the bilateral orbitofrontal and nucleus accumbens). Signal intensity was averaged within each brain region. In the end, all regions were assigned to one of seven well-known resting-state subnetworks^4^ based on maximum overlap: the default-mode network (DMN), fronto-parietal network (FPN), dorsal attention network (DAN), ventral attention network (VAN), visual, sensorimotor network (SMN); all deep grey matter regions were grouped into one separate subnetwork. Of all regions that were classified as the ‘limbic network’, only two regions showed sufficient signal. Thus, this network was removed from further analysis, leaving 7 networks and 190 brain regions per participant.

### Functional network analysis: subnetwork assignment

**Figure 1:**
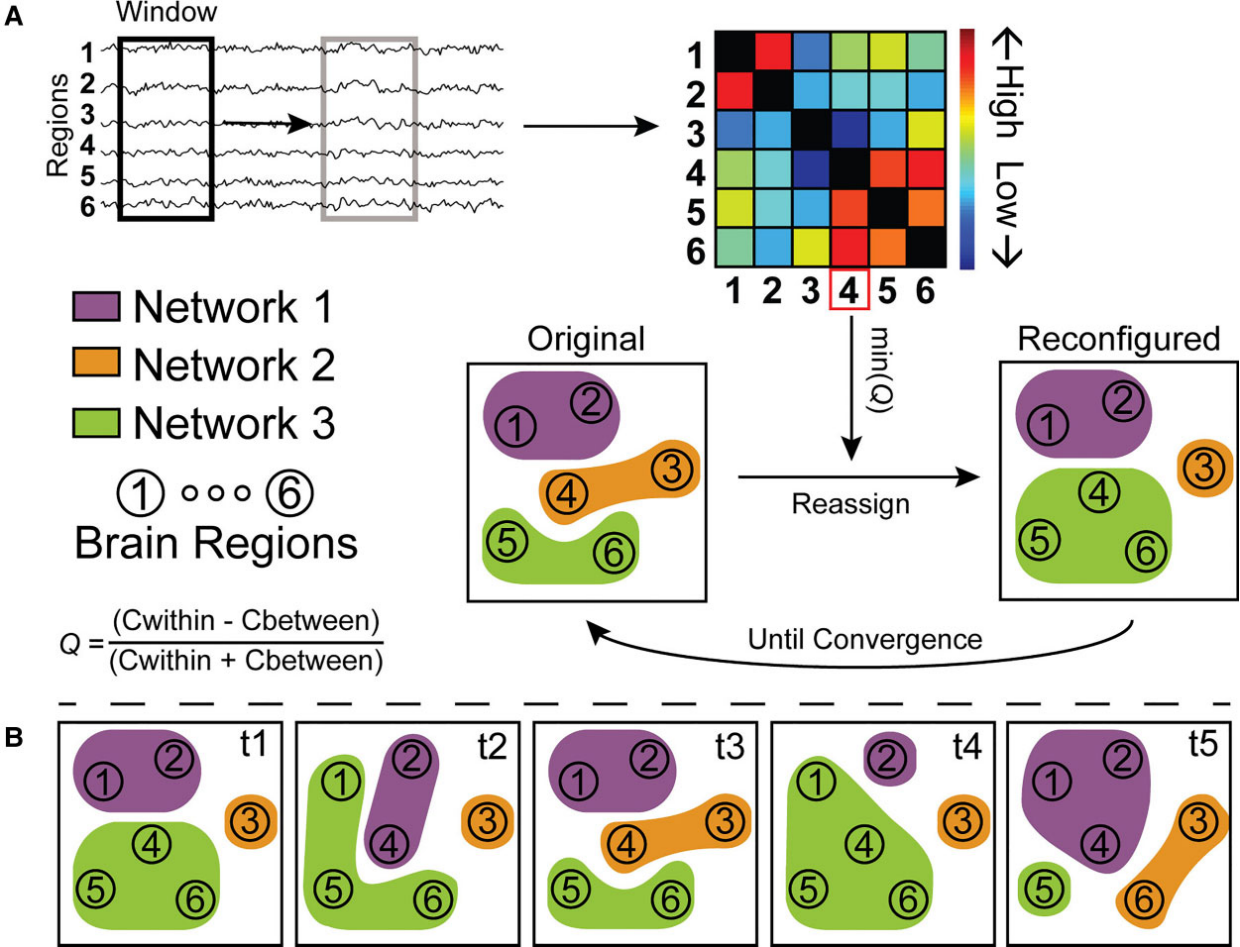
**A simplified illustration of the assignment of brain regions to networks and quantification of reconfiguration dynamics as used in this study.** (**A**) The pre-processed fMRI data was cut into smaller overlapping windows and connectivity was calculated between all regions within that window, resulting in a connectivity matrix for each window. Initially, the assignment of brain regions to networks is based on literature-derived networks, but this is iteratively updated by identifying the region with worst assignment using the original assignment, min(*Q*), and reassigning it to the network with which assignment quality would be maximized. This iterative reassignment is performed until the same regions is selected to have worst assignment two times in a row, signalling convergence. (**B**) Then, we could quantify network reconfiguration over time (from *t1* to *t5* in this example) using four measures. Promiscuity quantifies how many networks a region was assigned to, e.g. 2/3 for *region 1* and 3/3 for *region 4*. Flexibility quantifies how many times a region was reassigned over time, e.g. (4/4=)1 for *region 1* and 1/4 for *region 6*. Cohesion quantifies how many of these flexible reconfigurations are made together with another region, e.g. *region 1* and *region 4* switch assignment together from *t4* to *t5*. Finally, disjointedness quantifies how many reconfigurations are made independently, all other switches in this example are independent switches.

After deriving time-series, functional connectivity was determined using correlation analysis for a range of windows within each individual time-series to assess the dynamic network reconfiguration of functional networks. We used a sliding-window approach^39^ using a window of 60 s and a step-size of 10 s (yielding 27 windows) as has been suggested^40^ and as this size was found to capture the full range of dynamic network reconfiguration.^41^ Within each window, connectivity strength was calculated on a patient level between all regions using Fisher *r*-to-*z* transformed Pearson correlations (made absolute). Then, for each window, subnetwork assignment was iteratively re-evaluated using the assignment quality (*Q*), which was defined as the average connectivity strength of region *i* to other regions within the same assigned network (*C*_within_) minus the average connectivity strength to all remaining regions (*C*_between_) divided by the sum of the two; i.e. *Q_i_* = (*C*_within_ − *C*_between_)/(*C*_within_ + *C*_between_). In each iteration, (a) the brain region showing the worst assignment quality of the entire network was identified and (b) reassigned to the subnetwork it connects to most strongly (see Fig. 1A) using an in-house written script in MATLAB 2020b (Natick, Massachusetts, USA) that is accessible on GitHub (https://github.com/taabroeders/Recon_Dyn_MS/blob/main/CommunityDetection.m) This was repeated until the same brain region was selected in two successive iterations, indicating that further optimization was not possible.

### Patterns of dynamic network reconfiguration

Based on the dynamic subnetwork assignment, patterns of how regions were reassigned between subnetworks during the scan were first described using the terms ‘promiscuity’ and ‘flexibility’ based on the Dynamic Graph Metrics toolbox (see Fig. 1B).^42^ Promiscuity signifies the number of subnetworks a node participates in across all windows. Flexibility describes the number of reconfigurations a node makes, regardless of which subnetwork a region switches to or from. These two metrics were determined for each brain region and averaged over all regions initially assigned to the same subnetwork. Subsequently, the number of reconfigurations (i.e. flexibility) can be further differentiated using the terms ‘cohesion’ and ‘disjointedness’. Cohesion describes the number of times a node reconfigures from one subnetwork to another subnetwork together with another node (i.e. a mutual switch), whereas disjointedness describes the number of times a node switches between subnetworks individually (i.e. an independent switch). These parameters were used to discern whether either of the two best describes a change in flexibility, thus were treated as *post hoc* explorations beyond effects of flexibility. All reconfiguration parameters were quantified per brain region and averaged over all regions that belong to the same network, resulting in seven values per participant. These values were transformed to *z*-scores based on the distribution of HCs at each time-point, to correct for the scanner upgrade, and these *z*-scores were averaged over all networks to represent global dynamics.

### Validation analyses

Recent work has highlighted the importance of additional validation steps of dynamic analyses using null models, to evaluate whether or not effects are due to random noise or static connectivity changes. A null-distribution data was created by phase-randomization of the original time-series after Fourier-transformation,^43^ after which the entire dynamic pipeline was performed. The code for phase-randomization is available on GitHub (https://github.com/taabroeders/Recon_Dyn_MS/blob/main/Generate_surrogate.m). This process was repeated and analysed across 50 randomization runs, the average network reconfiguration metrics over all reconfiguration runs were calculated per subnetwork per participant to be used as null model comparisons. In addition, the effects of window size and shape were explored by calculating dynamic reconfiguration parameters using a shorter window-size of 44 s (as has been used previously^14^), as well as using a tapered instead of a square window shape by calculating weighted correlation coefficients using a Gaussian shape (*σ* = 3 TR).^44^

### Statistical analysis

Statistical analyses were performed in IBM SPSS version 26 (Armonk, NY, USA). First, ANOVAs and χ^2^ tests were performed to compare baseline clinical and demographic variables. Next, linear mixed models were used to compare baseline promiscuity and flexibility between cognitive groups (HC, CP, MCI and CI), correcting for age, sex and education. These analyses were primarily investigated across all networks (i.e. globally) and only considered statistically significant if the main effect survived correction for performing two comparisons using Bonferroni. As flexibility can be further differentiated using cohesion and disjointedness, these were tested between groups *post hoc* if flexibility was significant and used instead of flexibility in all further analyses. Bonferroni correction was applied over these two measures as well and if only one of these two measures showed a significant difference, only that one was investigated further instead of flexibility. When global effects were found, network-specific effects were investigated by performing the linear mixed models and correcting the main effects for performing multiple comparisons across all seven networks using Bonferroni. Additional validation analyses were performed by comparing significant differences between CI and CP patients using follow-up data, controlling for surrogate data and by further scrutinizing the results using different sliding-window parameters. Longitudinal changes were explored in cognitively declining and cognitively stable multiple sclerosis patients relative to HCs using linear mixed models, but only for those measures of reconfiguration dynamics that differed between CI and CP patients at baseline (i.e. cognitively relevant). Cognitively relevant dynamic reconfiguration parameters of multiple sclerosis patients were also correlated to average cognition, individual cognitive domains, EDSS score, fatigue and measures of structural damage (i.e. white matter FA, grey matter volume and lesion volume), using partial correlation coefficients corrected for age, sex and education.

Normality was checked using Kolmogorov–Smirnov test and histogram inspection, *P*-values <0.05 were considered statistically significant. The level of education was based on the highest level of education attained and was binarized for analyses (higher professional education yes/no). All reported *P*-values are corrected for performing multiple comparisons unless specifically indicated (i.e. *p*_uncorr_).

### Data availability

Anonymized data, not published in the article, will be shared on reasonable request from a qualified investigator.

## Results

### Demographic and clinical characteristics

Baseline demographics and clinical characteristic of the participants are summarized in Table 1. In the multiple sclerosis group, 134 (58.3%) were classified as CP (99 women; mean age 46 ± 10 years), 42 (18.3%) as MCI (26 women; mean age 49 ± 13 years) and 54 (23.5%) as CI (31 women; mean age 50 ± 12 years). Groups slightly differed on age, sex and education (see Table 1); all analyses were corrected for these three variables. Longitudinally, 65 patients were classified as cognitively declining and 165 as cognitively stable, and these groups did not differ on age, sex and education (see Supplementary Table 1). In addition, the proportion of treated patients or treatment type was comparable between cognitive groups, both cross-sectionally and longitudinally. The proportion of cognitively declining and stable patients was similar for each (at baseline defined) cognitive group (*P* = 0.252).

**Table 1:** Demographic, clinical and brain volumetric sample characteristics

	Multiple sclerosis				Test-statistic	*P*-value
	HC (*N* = 59)	CP (*N* = 134)	MCI (*N* = 42)	CI (*N* = 54)		
Demographics						
Male, *n*	28 (47.5%)^CP^	35 (26.1%)^HC^	16 (38.1%)	23 (42.6%)	*X*^2^ = 10.165	**0**.**017**
Age, y	45.99 ± 9.92	46.06 ± 10.14	49.03 ± 12.69	50.36 ± 11.56	*F* = 2.696	**0**.**046**
Level of education^¥^	6 (3)^MCI,CI^	6 (2)^MCI^	4 (3)^HC,CP^	4 (3)^HC^	*F* = 4.995	**0**.**002**
Disease characteristics						
Symptom duration	–	13.80 ± 7.96^CI^	14.48 ± 7.56	17.63 ± 9.8^CP^	*F* = 4.067	**0**.**018**
Disease phenotype, RRMS/SPMS/PPMS	–	114^CI^/15/5^MCI^	31/4/7^CP^	34^CI^/13/7	*X^2^* = 15.713	**0**.**003**
Treatment, Yes, *n*	–	49 (47,1%)	19 (45,2%)	18 (33,3%)	*X^2^* = 1.523	0.467
First line, *n*	–	37 (75,5%)	19 (100%)	14 (77,8%)	*X^2^* = 5.619	0.060
IFB/COP/NA/Other	–	31/6/9/3	13/5/0/0	12/2/3/1	*X^2^* = 6.833	0.337
Clinical variables						
EDSS ^¥^	–	3 (1.5)	3 (1.5)	4 (2.75)^CP^	*F* = 8.701	**<0**.**001**
Cognitive function	0.07 ± 0.47^a^	−0.18 ± 0.47^a^	−1.01 ± 0.31^a^	−1.77 ± 0.70^a^	*F* = 173.518	**<0**.**001**
Longitudinal cognition, Stable/declining	–	102/32	28/14	35/19	*X^2^* = 2.757	0.252
Fatigue (CIS-20)	–	72.47 ± 26.6	70.08 ± 26.8	79.8 ± 22.3	*F* = 1.315	0.272
Brain volume						
NDGMV (mL)	62.71 ± 3.47^a^	58.71 ± 4.89^a^	55.80 ± 6.01^a^	52.07 ± 7.49^a^	*F* = 40.371	**<0**.**001**
NCGMV (L)	0.78 ± 0.05^MCI,CI^	0.77 ± 0.04^CI^	0.75 ± 0.05^HC,CI^	0.72 ± 0.06^a^	*F* = 17.975	**<0**.**001**
Lesion volume (mL)	–	11.83 (9.18)^CI^	16.77 (13.69)^CI^	23.30 (17.78) ^a^	*F* = 16.459	**<0**.**001**

*Note.* All values represent means and standard deviations for the continuous variables but signify medians and the interquartile range (^¥^) or frequencies for categorical variables. Sample characteristics were compared between groups. The level of education was based on the highest level of education attained. Brain volumetric measures were transformed to litres (L) or millilitres (mL) for readability. Fatigue was assessed in a subset of participants (CP/MCI/CI: N = 64/24/35). *Post hoc* pairwise comparisons were Bonferroni corrected and *P*-values below 0.05 after correction were depicted in bold (^a^ = significantly different from all other groups, ^HC^ = significantly different from HC, ^CP^ = significantly different from CP, ^MCI^ = significantly different from MCI, ^CI^ = significantly different from CI). HC = healthy control, CP = cognitively preserved, MCI = mild cognitive impairment, CI = cognitive impairment, NDGMV = normalized deep grey matter volume, NCGMV = normalized cortical grey matter volume.

### Cross-sectional analyses

#### Global network reconfiguration

##### Promiscuity

**Figure 2:**
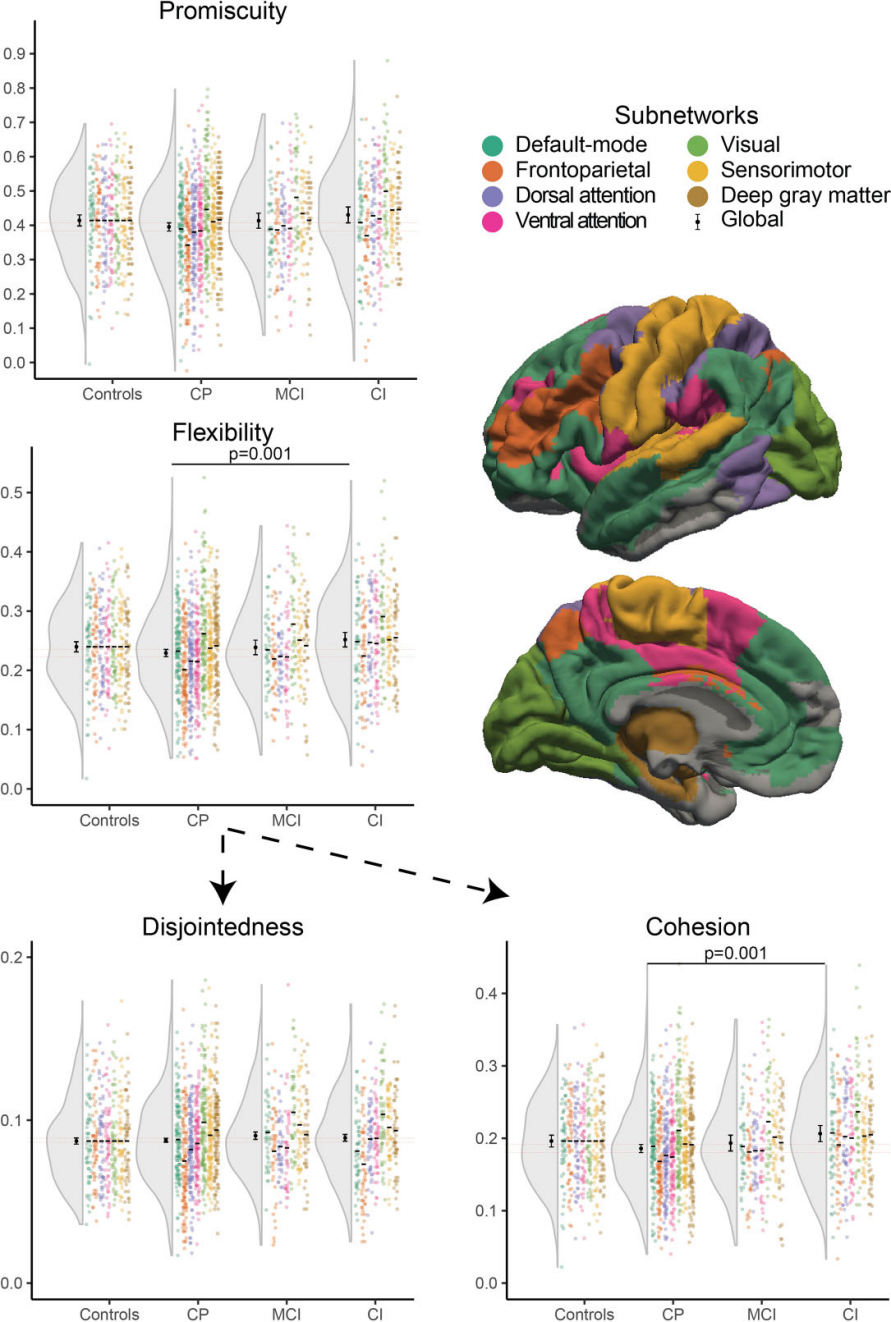
**Network reconfiguration dynamics per group at baseline.** Global flexibility (*P* = 0.001) was higher in CI patients compared with preserved (CP) patients, showing that brain regions switch more frequently between resting-state networks. Cohesion (*P* = 0.001) was particularly increased in CI patients compared with preserved patients and not disjointedness, indicating that the increased reconfigurations particularly occurred for pairs of brain regions (i.e. mutual switches). MCI patients showed intermediate dynamics. This effect does not seem to be specific to a network, but rather general across the whole brain. The coloured points indicate dynamics of each participant per network and the distribution over all networks is represented to the left of them, within that distribution the mean and confidence interval of global values are depicted. The horizontal dotted lines represent the confidence interval of global measures for CP patients, included for readability.

A main effect for cognitive group was seen when not correcting for multiple comparisons, driven by a higher promiscuity in CI compared with CP patients (*β* = 0.263, 95% CI = [0.070, 0.457], *P* = 0.008). However, this main effect did not survive multiple comparison correction [*F*(3,282) = 2.671, *P* = 0.096; see Table 2 and Fig. 2].

**Table 2:** Baseline reconfiguration dynamics

	Mean (±SD)				Main: group		CI versus CP	
	HC (*N* = 59)	CP (*N* = 134)	MCI (*N* = 42)	CI (*N* = 54)	*F*	*P*-value	*β* (95% CI)	*P*-value
Global effects								
Promiscuity	0.00 (±0.52)	−0.16 (±0.59)	−0.01 (±0.60)	0.14 (±0.71)	2.67	0.096	0.26 (0.07, 0.46)	**0**.**008**
Flexibility	0.00 (±0.48)	−0.15 (±0.52)	−0.02 (±0.57)	0.17 (±0.65)	3.72	**0**.**024**	0.29 (0.11, 0.46)	**0**.**001**
Flexibility type								
Cohesion	0.00 (±0.55)	−0.18 (±0.58)	−0.05 (±0.63)	0.18 (±0.73)	3.70	**0**.**024**	0.32 (0.12, 0.51)	**0**.**001**
Disjointedness	0.00 (±0.29)	0.02 (±0.30)	0.13 (±0.30)	0.08 (±0.30)	1.60	0.378	0.05 (−0.05, 0.14)	0.334
Network effects								
Cohesion								
DAN	0.00 (±1.00)	−0.35 (±1.03)	−0.24 (±0.78)	0.11 (±1.16)	2.80	0.280	0.42 (0.09, 0.75)	**0**.**014**
DMN	0.00 (±1.00)	−0.13 (±0.95)	−0.13 (±0.86)	0.20 (±1.16)	1.19	1.000	0.28 (−0.04, 0.60)	0.084
FPN	0.00 (±1.00)	−0.50 (±0.99)	−0.27 (±1.01)	−0.10 (±1.12)	3.69	0.084	0.39 (0.06, 0.720)	**0**.**021**
SMN	0.00 (±1.00)	−0.07 (±0.89)	0.10 (±0.81)	0.12 (±0.78)	0.60	1.000	0.15 (−0.13, 0.43)	0.283
DGM	0.00 (±1.00)	−0.09 (±1.12)	−0.04 (±1.21)	0.16 (±1.13)	0.27	1.000	0.15 (−0.21, 0.51)	0.408
VAN	0.00 (±1.00)	−0.39 (±0.97)	−0.24 (±1.20)	0.07 (±1.09)	2.90	0.245	0.43 (0.09, 0.76)	**0**.**013**
Visual	0.00 (±1.00)	0.26 (±1.24)	0.48 (±1.22)	0.72 (±1.29)	3.05	0.203	0.42 (0.03, 0.81)	**0**.**037**

*Note.* Global flexibility differed between groups at baseline. When further scrutinizing the types of flexible switches, group differences were solely found for cohesion (i.e. mutual switches) and not disjointedness (independent switches). Most notably, CI patients showed greater global reconfiguration dynamics compared with CP patients, with HCs and MCI patients showing intermediate dynamics. Reconfiguration dynamics did not seem to be specific to a particular network. The *z*-scores were based on the distribution of HCs within each networks and global dynamics represented the average over all networks. The reported *P*-values for the main group effects were corrected for multiple comparisons using Bonferroni correction and *P*-values below 0.05 after correction were depicted in bold. Subnetworks: DAN, DMN, FPN, SMN, deep grey matter, VAN and visual network.

##### Flexibility

A main effect for cognitive group was found [*F*(3,282) = 3.719, *P* = 0.024]. CI patients showed increased global flexibility compared with CP patients [*β* = 0.286, 95% CI = (0.113, 0.459), *P* = 0.001], which indicates that brain regions changed the subnetwork they participated in more often in CI patients. No other differences were observed between cognitive groups.

##### Cohesion and disjointedness

Based on the flexibility results, cohesion and disjointedness were explored as well. A difference in global cohesion strength was found between cognitive groups [*F*(3,282) = 3.704, *P* = 0.024; see Table 2], as CI patients showed higher global cohesion than CP patients [*β* = 0.319, 95% CI = (0.124, 0.514), *P* = 0.001]. No other differences were observed between cognitive groups for cohesion. In addition, no global effect for cognitive status was found for disjointedness [*F*(3,282) = 0.905, *P* = 0.876]. Thus, brain regions changed assignment more often together with other regions (i.e. mutual switches) and not independently in CI patients. In additional exploratory analyses the characteristics of cohesion were further investigated, showing more frequent mutual switches between regions that normally do not switch together in CI compared with CP (see Supplementary material).

For all aforementioned analyses that showed significant group differences, age was a significant covariate but sex and education were not.

The dynamic reconfigurations of a full resting-state scan have been visualized for a single representative HC (Video 1). In addition, the reconfigurations have been visualized for the participant that showed the lowest number of mutual switches (a CP patient) and the participant showing the highest number of mutual switches (a CI patient; Video 2).

#### Subnetwork-specific reconfiguration

Based on the global findings, subnetwork-specific effects were only investigated for cohesion (see Table 2). No subnetwork-specific main effects of group were found after correcting for multiple comparisons (all *p* > 0.084). The DAN [*F*(3,282) = 2.803, *p*_uncorr_ = 0.040], FPN [*F*(3,282) = 3.687, *p*_uncorr_ = 0.012], VAN [*F*(3,282) = 2.900, *p*_uncorr_ = 0.035] and visual network [*F*(3,282) = 3.049, *p*_uncorr_ = 0.029] did show group differences in cohesion without correcting for multiple comparisons, with fixed effects indicating increased cohesion for CI compared with CP patients in the DAN [*β* = 0.416, 95% CI = (0.086, 0.747), *P* = 0.014], FPN [*β* = 0.391, 95% CI = (0.059, 0.724), *P* = 0.021], VAN [*β* = 0.425, 95% CI = (0.092, 0.759), *P* = 0.013] and visual network [*β* = 0.417, 95% CI = (0.026, 0.807), *P* = 0.037]. In addition, cohesion was reduced in CP patients compared with HCs in the DAN [*β* = −0.335, 95% CI = (−0.653, −0.018), *P* = 0.039], FPN [*β* = −0.481, 95% CI = (−0.800, −0.162), *P* = 0.003] and VAN [*β* = −0.357, 95% CI = (−0.678, −0.037), *P* = 0.029].

### Validation analyses

#### Follow-up cross-sectional analysis

Similarly to the baseline analyses, cohesion based on the follow-up scans was higher in CI compared with CP patients defined on the follow-up cognitive tests [*β* = 0.322, 95% CI = (0.534, 0.109), *P* = 0.003).

#### Null model

The global effects for cohesion derived from the empirical data were still significantly increased in CI compared to CP patients when additionally controlling for global cohesion values derived from randomized data [*β* = 0.112, 95% CI = (0.023, 0.200), *P* = 0.013], indicating that this measure picked up more than just noise or static connectivity differences.

#### Window size and shape

Looking at the effects of using a shorter window size [*β* = 0.297, 95% CI = (0.104, 0.491), *P* = 0.003] or a Gaussian window shape [*β* = 0.281, 95% CI = (0.086, 0.476), *P* = 0.005], global cohesion remained increased in CI compared with CP patients. Together, these analyses support the validity of global cohesion.

### Longitudinal analyses

**Figure 3:**
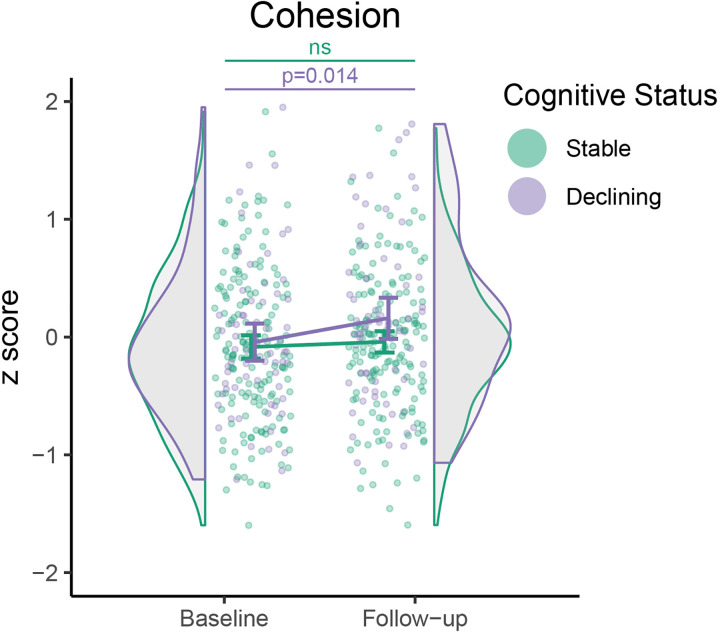
**Longitudinal change in cohesion for patients.** Cohesion increased over time in declining patients relative to HCs (*P* = 0.014), but not in stable patients (*P* = 0.390). HC values are not shown, as patient scores were normalized relative to the distribution of HCs at each time-point.

Based on cross-sectional findings, only global effects for cohesion were investigated. Global cohesion of multiple sclerosis patients increased over time relative to HCs [*F*(1,226) = 6.549, *P* = 0.011] but only in cognitively declining patients [*β* = 0.204, 95% CI = (0.042, 0.365), *P* = 0.014] and not in cognitively stable patients [*β* = 0.044, 95% CI = (−0.057, 0.146), *P* = 0.390; see Fig. 3). No differences were observed between the two groups [*F*(4,226) = 2.715, *P* = 0.101] not at baseline [*β* = 0.006, 95% CI = (−0.171, 0.184), *P* = 0.944] or at follow-up [*β* = 0.153, 95% CI = (−0.024, 0.330), *P* = 0.090].

### Correlations with other clinical measures

#### Clinical performance

In multiple sclerosis patients, lower average cognition at baseline was related to higher cohesion (*r* = −0.182, *P* = 0.007). This holds true after additionally adjusting for EDSS (*r* = −0.140, *P* = 0.036), whereas no relation was found between EDSS and cohesion (*r* = 0.118, *P* = 0.078) after correcting for average cognition, suggesting that reconfiguration dynamics is especially important for cognition and not physical impairment. In particular, higher cohesion was related to worse verbal memory (*r* = −0.156, *P* = 0.022), IPS (*r* = −0.202, *P* = 0.003) and working memory (*r* = −0.163, *P* = 0.017) in multiple sclerosis patients. Finally, for fatigue, no relationship with cohesion was observed (*r* = 0.014, *P* = 0.874).

#### Structural damage

In multiple sclerosis patients, higher cohesion was related to lower normalized deep grey matter (*r* = −0.183, *P* = 0.006) and cortical grey matter volume (*r* = −0.162, *P* = 0.015) and FA of white matter tracts (*r* = −0.143, *P* = 0.032). In addition, more lesion volume in multiple sclerosis patients is related to higher global cohesion (*r* = 0.176, *P* = 0.008).

## Discussion

In this study, multiple sclerosis patients with cognitive impairment showed more dynamic reconfigurations of the functional network compared with CP patients. Subnetworks were reconfigured more frequently, and this increase was specifically salient for pairs of brain regions being reconfigured in unison (i.e. mutual switches). These findings were found across the entire brain and did not seem to be specific for one particular subnetwork. In addition, mutual switches increased over 5 years in patients that showed cognitive decline over those years. More extensive dynamic network reconfiguration in multiple sclerosis patients related to more severe structural damage to the white and grey matter and worse cognitive performance on IPS, verbal memory and working memory.

CI patients showed globally higher reconfiguration dynamics (i.e. higher flexibility) compared with patients that were unimpaired, as brain regions were reconfigured more frequently across subnetworks. Peripheral brain regions are reconfigured continuously and precisely to promote integration across subnetworks,^17^ but the network must also dedicate a rigid core of regions to focus and to remain stable during a cognitive task.^41^ Thus, there is a delicate balance between too much and too little reconfigurations and the increased subnetwork reconfigurations in CI multiple sclerosis patients could indicate that reconfigurations became more erratic. This implies that the functional network had become less stable.^22^ These results shed new light on previous findings that showed that the functional network in CI multiple sclerosis patients seems ‘stuck’ in a state of high DMN connectivity,^12,14,16,45^ suggesting that this ‘stuckness’ co-occurs with more dynamic integration through the reconfiguration of brain regions across subnetworks. Although this might seem counterintuitive, the DMN consists of many central or ‘hub’ regions that are strongly involved in integrating information across the network to more peripheral brain regions.^46,47^ Peripheral regions, however, are mainly involved in integrating information across subnetworks through dynamic reconfiguration across subnetworks.^48^ Thus, both these findings might reflect a network with more intense integration of information, as the DMN remains stuck in a highly connected state, where peripheral regions might reconfigure more than normal. As non-hub regions are more widely dispersed across the network,^46^ this might explain our lack of network-specific effects, although this hypothesis needs validation in future work. Finally, in theory, more extensive reconfiguration dynamics could require sustained effort and burden on the network, which might play an important role in the development of fatigue in multiple sclerosis,^49^ but no relationship between cohesion and fatigue was observed in this study.

Beyond more frequent reconfigurations, switches between subnetworks that featured pairs of brain regions (i.e. mutual switches) were particularly heightened in CI, which further increased in patients that showed cognitive decline. Mutual switches have been studied before in the healthy brain and this type of reconfiguration was regarded as a marker of coordinated changes in subnetwork organization.^20,50^ This seemingly contrasts the notion of more erratic reconfigurations across subnetworks in multiple sclerosis. However, this mutual switching (i.e. cohesion) could also be considered as an increased viscosity of the network. In support, we performed additional analyses exploring the underlying pattern of mutual switches, showing that these actually occurred between regions that normally do not switch together as frequently, in line with the concept of an increased viscosity. These mutual switches further increased in patients that showed longitudinal cognitive decline. Other longitudinal studies investigating network changes related to cognitive decline are scarce. One recent study performed in the same cohort has indicated that an initially disturbed functioning of the VAN might precede both DMN disturbance and more pronounced cognitive impairment, as longitudinal changes in VAN centrality were exclusively observed in stable patients and no changes were observed in declining patients.^24^ Another study in early multiple sclerosis also found that the static organization of the functional network alone did not relate to cognitive decline.^51^ These results suggest that the longitudinal cognitive decline might not be well-reflected by static functional network changes alone. Instead, the interplay between structure and function was found to be relevant to cognitive decline in early multiple sclerosis.^51^ Although we did not formally evaluate the interplay between structure and function in this study, the dynamic network reconfiguration parameters in this study related strongly to common markers of structural damage in multiple sclerosis. Future work could, therefore, investigate the role of structural network changes on dynamic network parameters further. In addition, structural damage might induce noise in functional quantifications which should also be explored further.

The origins of such an increase in dynamic reconfiguration in multiple sclerosis remains elusive but could relate to an altered balance between excitation and inhibition and regulatory functions between networks. For instance, multiple sclerosis features an extensive loss of inhibitory neurons, which are thought to have more control on overall network functioning compared with excitatory neurons.^52^ As such, the imbalance between excitatory and inhibitory control in multiple sclerosis^53^ might have led to disinhibition of the DMN. Accordingly, in healthy individuals the DMN and visual network show a negative correlation, but this was lost in CI multiple sclerosis patients.^14^ Subsequently, DMN disinhibition might result in more erratic network reconfigurations, possibly related to impaired VAN functioning, a network known to be crucial for managing network balance in the brain.^24^ On top of these functional effects, continuing damage to structural pathways could result in an increasing constraint of functional connectivity,^51,54^ which could drive regions to switch together even though they normally would not. However, future work is needed to better comprehend the entire structural and functional cascade of events leading to such a disruption in whole-brain network dynamics.

Reconfiguration dynamics of the whole brain were more strongly related to cognitive than physical impairment in multiple sclerosis. CI patients showed more physical impairment than CP patients, but the amount of physical impairment did not relate to global reconfiguration dynamics after correcting for the amount of cognitive impairment. Although there is some association between physical and cognitive impairment, this is not always the case in multiple sclerosis.^55^ Previous (static) functional network studies have reported distinct patterns of functional connectivity changes related to either motor or cognitive symptoms,^56^ and the SMN seems to play a particularly important role for physical impairment only.^25^ More research is needed to understand whether functional network dynamics affects motor performance as well, since previous studies have been limited in size.^57^ In addition, previous work has shown some promise with more advanced measures of disability compared to EDSS, especially with more complex tasks of hand functioning, which seem to have more cognitive circuitry involved compared with walking tests.^58^ Looking at individual cognitive domains, relations with IPS, working memory and verbal memory were most pronounced, all of whom have been related to dynamic connectivity changes in multiple sclerosis before.^29,59–61^ These domains are also known to be commonly impacted in multiple sclerosis,^62^ and to be related to a wide variety of brain regions,^27,63^ which could explain these results.

Some limitations should be addressed. First, we only studied established multiple sclerosis and future studies could investigate the first phase of the disease to capture the earliest effects and potential compensatory processes as a result of structural damage. Furthermore, although cognitive decline was identified over 5 years from baseline to follow-up, the decline in the current sample was relatively mild so even longer-term assessment is needed. The slow accumulation of cognitive impairment is best measured over long time windows, thus larger effect sizes are commonly seen cross-sectionally. This could also be the reason for the observed sex differences between cognitive groups only at baseline. Alternatively, sex effects in longitudinal decline might mainly play a role early in the disease, which was not covered by our cohort. In addition, even though functional connectivity was found to be relatively stable across scanners,^64^ there was a major update between time-points, which is why time-point-specific *z*-scores were used based on HCs. In dynamic network studies, it is important to look for effects of spurious dynamics, which is why sensitivity analyses were added to show that the current methods capture network reconfiguration that cannot solely be explained by the static organization of the network. Finally, future studies are needed to study task-based paradigms to further reveal whether additional information on dynamic reconfigurations can be captured during active cognitive processing in multiple sclerosis.^48^

## Conclusion

Multiple sclerosis patients with cognitive impairment exhibited a more unstable network, i.e. brain regions switched between subnetworks more often. This reduced network stability worsened longitudinally in cognitively declining patients only. These results suggest that the functional network reconfigurations become more erratic over time as patients transition towards more severe cognitive impairment in multiple sclerosis, thus supporting the hypothesis that the multiple sclerosis network progressively destabilizes. Future studies are now required to further elucidate the specific pathological mechanisms leading to such a network destabilization over time.

## Supplementary material

Supplementary material is available at *Brain Communications* online.

## Supplementary Material

fcac095_Supplementary_Data

## Abbreviations

CI =: cognitively impaired
CP =: cognitively preserved
DAN =: dorsal attention network
DMN =: default-mode network
EDSS =: Expanded Disability Status Scale
FPN =: fronto-parietal network
HC =: healthy control
IPS =: information processing speed
MCI =: mildly cognitively impaired
rs-fMRI =: resting-state functional MRI
SMN =: sensorimotor network
TE =: echo time
TR =: repetition time
VAN =: ventral attention network
