# Supplementary material for: A more unstable resting-state functional network in cognitively declining multiple sclerosis
Source: Brain Commun. 2022 Apr 12;4(2):fcac095. doi: 10.1093/braincomms/fcac095 (PMC9128379; doi:10.1093/braincomms/fcac095)
Supplement: fcac095_Supplementary_Data [file fcac095_Supplementary_Data.docx]

# Supplementary Material

To investigate how brain regions reconfigure in unison, the number of times each pair of brain regions switched in unison was quantified for all participants. In healthy controls, the most often co-switching regions included the thalamus (mean=6.19), ventral area 23 (mean=4.90), hippocampus (mean=4.68), dorsal area 23 (mean=4.22) and caudal area 23 (mean=4.05). Interestingly, of the twenty pairs that showed most mutual switches on average, eighteen were contralateral pairs of brain regions and the two that were not a contralateral pair were spatially adjacent and known to be involved in related functions. This highlights that mutual switches mostly happen with functionally related brain-regions.

Subsequently, we explored whether the increase in cohesion in CI multiple sclerosis patients was most prominent in these functionally related brain regions, or alternatively occurred in brain regions that usually do not switch together frequently. Firstly, we confirmed that the mean number of mutual switches over all pairs of brain regions (HC-mean=0.171) was different between the four groups (*F*(3,282)=3.286, *p*=0.021) and higher in CI compared to CP patients (β=0.025, *95% CI*=[0.008, 0.043], *p*=0.005), similarly to the results for cohesion. When only comparing the mean number of mutual switches of pairs of brain regions that belong to the same subnetwork (HC-mean=0.518, i.e. these switch together relatively frequently) there is no main group effect (*F*(3,282)=1.605, *p*=0.188) and no difference between CI and CP (β=0.040, *95% CI*=[-0.007, 0.088], *p*=0.100). However, when only comparing the mean number of mutual switches between pairs of brain regions that *do not* belong to the same subnetwork (HC-mean=0.108, i.e. these switch together less frequently) there is a difference between groups (*F*(3,282)=4.097, *p*=0.007) and the number was higher in CI compared to CP patients (β=0.023, *95% CI*=[0.009, 0.036], *p*=0.001). Thus, this suggests that the mutual switches particularly increased between brain regions that usually do not switch together frequently.

**Supplementary Table 1.** Demographic and disease characteristics of longitudinal groups

|  |  | Multiple Sclerosis | |  |  | |
| --- | --- | --- | --- | --- | --- | --- |
|  | HC (N=59) | Stable (N=165) | Declining (N=65) |  | Test-statistic | p-value |
| **Demographics** |  |  |  |  |  |  |
| Male, n | 28 (47.5%) | 51 (30.9%) | 23 (35.4%) |  | *X^2^*=5.212 | 0.074 |
| Age, y | 45.99 ± 9.92 | 46.75 ± 11.08 | 49.80 ± 10.88 |  | *F*=2.384 | 0.094 |
| Level of education^¥^ | 6 (3) | 5 (2) | 4 (3) |  | *F*=3.000 | 0.051 |
| **Disease characteristics** |  |  |  |  |  |  |
| Symptom duration | - | 14.3 ± 8.4 | 16.1 ± 8.5 |  | *F*=1.935 | 0.166 |
| Disease phenotype,  RRMS/SPMS/PPMS | - | 142/14/9 | 37/18/10 |  | *X^2^*=23.018 | **<0.001** |
| Treatment,  Yes, n | - | 65 (39,4%) | 21 (32,3%) |  | *X^2^*=1.000 | 0.317 |
| First line, n | - | 52 (80,0%) | 18 (85,7%) |  | *X^2^*=0.342 | 0.559 |
| IFB/COP/NA/Other | - | 44/8/11/2 | 13/5/1/2 |  | *X^2^*=4.570 | 0.206 |

*Note.* All values represent means and standard deviations for the continuous variables, but signify medians and the interquartile range (^¥^) or frequencies for categorical variables. Sample characteristics were compared between groups. The level of education was based on the highest level of education attained. Brain volumetric measures were transformed to litres (L) or millilitres (mL) for readability. Fatigue was assessed in a subset of participants (HC/Stable/Declining: N = 29/85/38). HC = healthy control, NDGMV = normalized deep grey matter volume, NCGMV = normalized cortical grey matter volume.
